# Effect of Delayed Presentation on the Prognosis of Snakebite Victims at a Tertiary Care Hospital: The Golden Hour Matters

**DOI:** 10.7759/cureus.115910

**Published:** 2026-09-07

**Authors:** Vikash Chandra, Shivangini Srivastava, Mukul Sharma

**Affiliations:** 1 Department of Forensic Medicine and Toxicology, All India Institute of Medical Sciences, Raebareli, Raebareli, IND

**Keywords:** anti-snake venom, delayed presentation, envenomation, golden hour, mortality, snakebite

## Abstract

Introduction: Snakebite envenomation is one of the important public health problems in India, and many deaths and morbidity in rural areas are attributed to snakebite envenomation.

Objective: This study aimed to evaluate the impact of time from bite to hospital on clinical outcomes of snakebite patients admitted to a tertiary care centre in Uttar Pradesh.

Methodology: A cross-sectional observational study was conducted at All India Institute of Medical Sciences (AIIMS), Raebareli, for a period of one year, from March 2025 to March 2026. A total of 30 envenomated patients who were confirmed to have been bitten by snakes were included and divided into four groups: Group I (patients bitten within six hours), Group II (six to 12 hours), Group III (12-18 hours), and Group IV (18-24 hours). Severity of disease, systemic toxicity, anti-snake venom (ASV) requirements, complications, and mortality were assessed.

Results: The mean age was 28.0 years (range 9-70). Most patients were male (53.3%) and from low socioeconomic backgrounds (53.3%). Lower limb bites predominated (53.3%). Dyspnoea and drowsiness were universal (100%). Out of four deaths, one occurred in patients presenting within six hours and three deaths in patients presenting between 18 and 24 hours, with 75% mortality in the 18-24-hour group (3/4) versus 7.1% in the zero-to-six-hour group (1/14). Recovery rates were highest in the zero-to-six-hour group (71.4%).

Conclusion: Delayed hospital presentation is strongly associated with worse outcomes in snakebite envenomation. The "golden hour" principle is reaffirmed, and early ASV administration within six hours significantly improves survival.

## Introduction

India bears the world's highest burden of snakebite envenomation, with an estimated 46,000 deaths annually according to the Million Death Study [1]. The "Big Four" venomous species - the Indian cobra *(Naja naja)*, common krait *(Bungarus caeruleus)*, Russell's viper *(Daboia russelii)*, and saw-scaled viper *(Echis carinatus)* - account for the majority of clinically significant cases [2]. Venom effects range from neurotoxic paralysis to haemotoxic coagulopathy and cytotoxic tissue necrosis [3].

ASV is the only specific antidote, but its efficacy diminishes with time [4]. Once venom binds to target tissues, blocking neuromuscular junctions or triggering consumptive coagulopathy, the damage becomes largely irreversible [5]. Delayed presentation allows systemic toxicity to progress beyond the therapeutic window [5].

Systemic snake envenomation requires only one specific treatment, namely, anti-snake venom (ASV). The commonly available and routinely used ASV in India is polyvalent against the venom of the "big four" species, namely, *Naja naja* (Indian cobra), *Bungarus caeruleus* (common krait), *Daboia russelii* (Russell’s viper), and *Echis carinatus* (saw-scaled viper). Therefore, Indian polyvalent ASV does not provide protection against all snake envenomation, especially those not included in the immunisation cocktail, including from species of venom lacking in the mixture. The use of ASV is recommended when clinical and/or laboratory evidence of envenomation is present, and it is administered intravenously under proper medical supervision. Under the National Action Plan for Prevention and Control of Snakebite Envenoming, the government of India is trying to improve the accessibility and affordability of ASV by public-sector procurement and essential-drug programmes.

Snakebite incidences are high in rural India, and there are several factors that delay access to hospitals, such as poor transport infrastructure, financial constraints, reliance on traditional healers, harmful first-aid practices (tourniquets and incisions), and referral delays from peripheral facilities [6-8]. There is limited regional data on presentation time and outcomes in Uttar Pradesh. This study was undertaken to estimate the impact of bite-to-hospital time on the prognosis at All India Institute of Medical Sciences (AIIMS), Raebareli.

## Materials and methods

Study design and setting

The current study is a cross-sectional observational study carried out in the Emergency Department of AIIMS Raebareli, Uttar Pradesh, India, from March 2025 to March 2026, for a period of one year. The Institutional Ethics Committee of AIIMS Raebareli issued approval (no. EC/NEW/INST/2024/UP/0572).

Study population

Patients with possible snake bites were screened for eligibility. Patients were admitted when they had a definite history of snakebite with clinical and/or laboratory findings consistent with envenomation. Patients with ≥1 month of age, a clear history of snakebite, and local or systemic signs of envenomation or laboratory abnormalities were included. Incomplete clinical information, or patients who were not clinically assessed and did not have evidence of envenomation, were not included in the study. Patients who did not stay in the study hospital after discharge were excluded from the analysis if no outcome data were available prior to discharge, and included if outcome data were obtained after discharge.

Data collection

Data were collected using a structured proforma and supplemented by review of clinical and treatment records. The variables recorded included demographic characteristics, socioeconomic status, relevant comorbidities, snakebite history, time of bite, time of hospital presentation, bite site, clinical manifestations, vital signs, and laboratory parameters, including 20-minute whole blood clotting time, complete blood count, and renal function tests. Information regarding the identified snake species, when available, was also recorded. Treatment-related variables included ASV administration and dose, ventilatory support, intensive care unit (ICU) admission, complications, and duration of hospitalisation. The final recorded outcome was categorised as complete recovery, death, or leaving against medical advice (LAMA).

Classification of snakebite and clinical severity

Patients were assessed at presentation for local and systemic manifestations of envenomation. Signs and symptoms that were considered to be included were local pain, swelling, blisters, bleeding, tissue injury, and systemic signs (ptosis, dysphagia, dysarthria, respiratory distress, drowsiness, and altered sensorium). Laboratory signs of envenomation, such as changes in clotting status and renal function, were also documented. The nature of the envenomation was determined by the predominant clinical and laboratory findings and, when available, by the species of snake identified.

Group according to time to presentation

Patients were divided into four groups based on the time gap between the time of snake bite and the time of presentation at the study hospital: Group I (0 to <6 hours), Group II (6 to <12 hours), Group III (12 to <18 hours), and Group IV (18 to <24 hours) (Table 1).

**Table 1:** Baseline characteristics according to time to hospital presentation

Characteristic	Group I <6 h (n = 14)	Group II 6–12 h (n = 10)	Group III 12–18 h (n = 2)	Group IV 18–24 h (n = 4)
Age, mean ± SD (years)	28.4 ± 13.1	30.7 ± 14.2	39.0 ± 18.4	43.5 ± 15.7
Male, n (%)	8 (57.1)	5 (50.0)	1 (50.0)	2 (50.0)
Female, n (%)	6 (42.9)	5 (50.0)	1 (50.0)	2 (50.0)
Any comorbidity, n (%)	3 (21.4)	2 (20.0)	1 (50.0)	1 (25.0)
Ptosis, n (%)	12 (85.7)	9 (90.0)	2 (100)	4 (100)
Dysphagia, n (%)	10 (71.4)	8 (80.0)	2 (100)	4 (100)
Dyspnoea, n (%)	14 (100)	10 (100)	2 (100)	4 (100)
Drowsiness, n (%)	13 (92.9)	10 (100)	2 (100)	4 (100)
Prolonged 20-min WBCT, n (%)	3 (21.4)	3 (30.0)	1 (50.0)	1 (25.0)
Abnormal renal function, n (%)	2 (14.3)	2 (20.0)	1 (50.0)	1 (25.0)

Outcome measures

Mortality within the hospital was the main outcome. Secondary outcomes were complete recovery, LAMA, clinical presentation, need and dose of ASV, ventilatory support, ICU admission, complications in hospital and hospital length of stay. The four presentation-time groups were compared for baseline characteristics: age, sex, presence of any comorbidities, clinical symptoms, laboratory parameters, and available information on the species of snake involved, to evaluate potential differences in baseline severity and other parameters which could impact outcome. The distribution of identified snake species according to presentation time is shown in Table 2.

**Table 2:** Distribution of identified snake species according to the presentation time

Identified snake species	Group I <6 h (n = 14)	Group II 6–12 h (n = 10)	Group III 12–18 h (n = 2)	Group IV 18–24 h (n = 4)
Bungarus caeruleus	5	4	1	1
Naja naja	4	3	1	1
Daboia russelii	2	2	0	1
Echis carinatus	1	0	0	0
Unidentified	2	1	0	1
Total	14	10	2	4

## Results

Demographics

The mean age was 28.0 years (range 9-75 years). The 18-30 years age group constituted the largest proportion (50.0%, n = 15), followed by <18 years (20.0%, n = 6), 31-45 years (13.3%, n = 4), 46-60 years (13.3%, n = 4), and 61-75 years (3.3%, n = 1). Males comprised 53.3% (n = 16) and females 46.7% (n = 14) (Table 3).

**Table 3:** Outcomes by time-to-presentation group (N = 30)

Presentation group	n	Death, n (%)	Complete recovery, n (%)	LAMA, n (%)
Group I (<6 h)	14	1 (7.1)	10 (71.4)	3 (21.4)
Group II (6–12 h)	10	0 (0.0)	9 (90.0)	1 (10.0)
Group III (12–18 h)	2	0 (0.0)	2 (100.0)	0 (0.0)
Group IV (18–24 h)	4	3 (75.0)	1 (25.0)	0 (0.0)
Total	30	4 (13.3)	22 (73.3)	4 (13.3)

Bite characteristics and pre-hospital care

Bites occurred more commonly at night (56.7%, n = 17) than during the day (40.0%, n = 12), while the timing was unrecorded in one case (3.3%, n = 1), and unknown/other (3.3%, n = 1). Most patients (53.3%, n = 16) belonged to low socioeconomic status, 26.7% (n = 8) to lower-middle, and 20.0% (n = 6) to upper-lower class. The majority traveled 15-20 km to reach hospital (73.3%, n = 22). First aid practices included ASV administration (86.7%, n = 26), faith healer consultation (10.0%, n = 3), and harmful incision at the bite site (3.3%, n = 1). Snake species was identified in 70.0% (n = 21) of cases.

Clinical presentation

Systemic symptoms were predominantly neurotoxic: dyspnoea (100.0%, n = 30), drowsiness (100.0%, n = 30), slurring of speech (90.0%, n = 27), ptosis (90.0%, n = 27), dysphagia (80.0%, n = 24), and vomiting (73.3%, n = 22). Local effects included pain at bite site (43.3%, n = 13), abdominal pain (50.0%, n = 15), local edema (23.3%, n = 7), and local bleeding (23.3%, n = 7). Blisters were present in 13.3% (n = 4), cellulitis in 6.7% (n = 2), and necrosis in 3.3% (n = 1). Figures 1-2 show examples of local tissue reactions.

**Figure 1:**
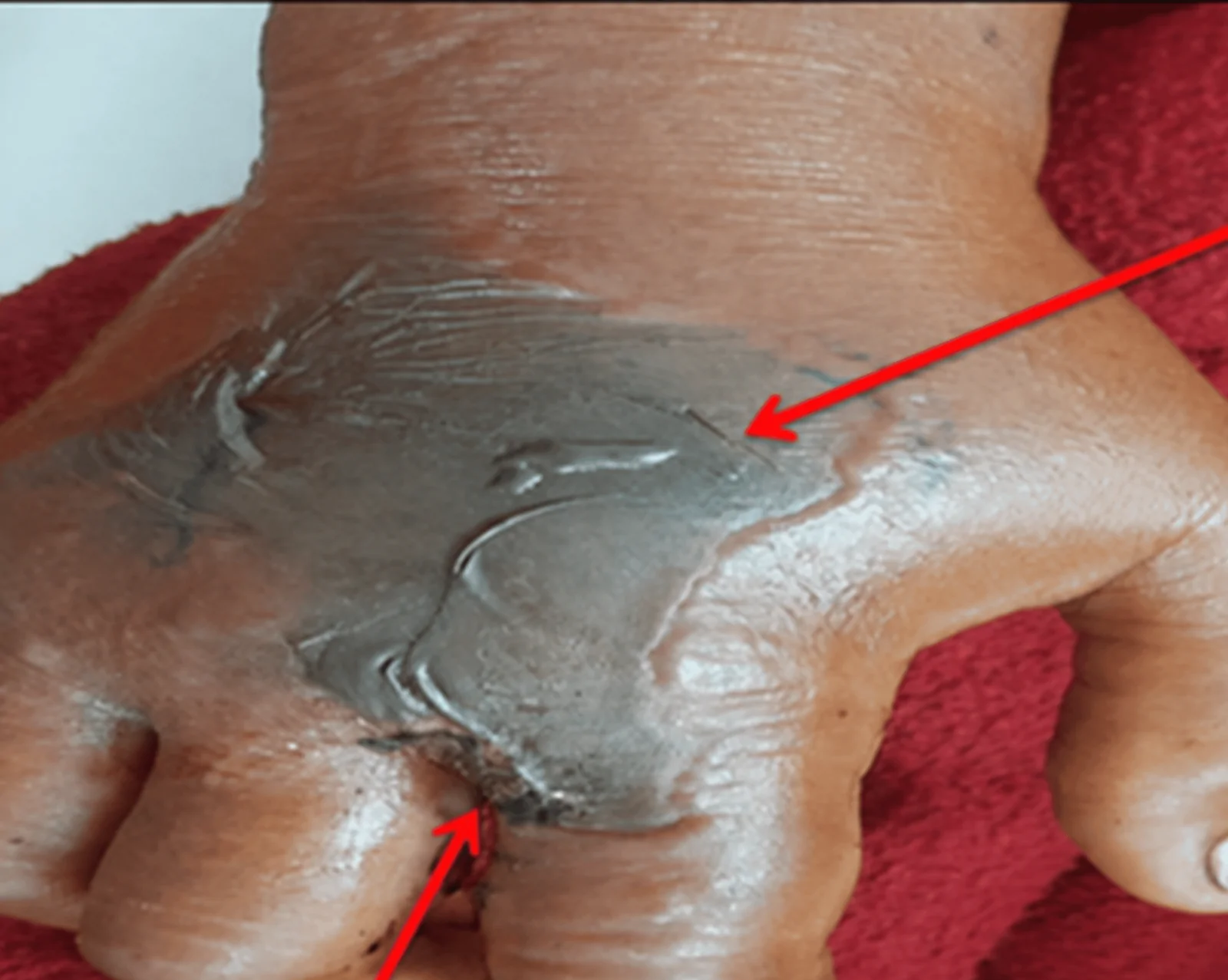
Extensive edema and local tissue reaction over the dorsum of the hand at the bite site.

**Figure 2:**
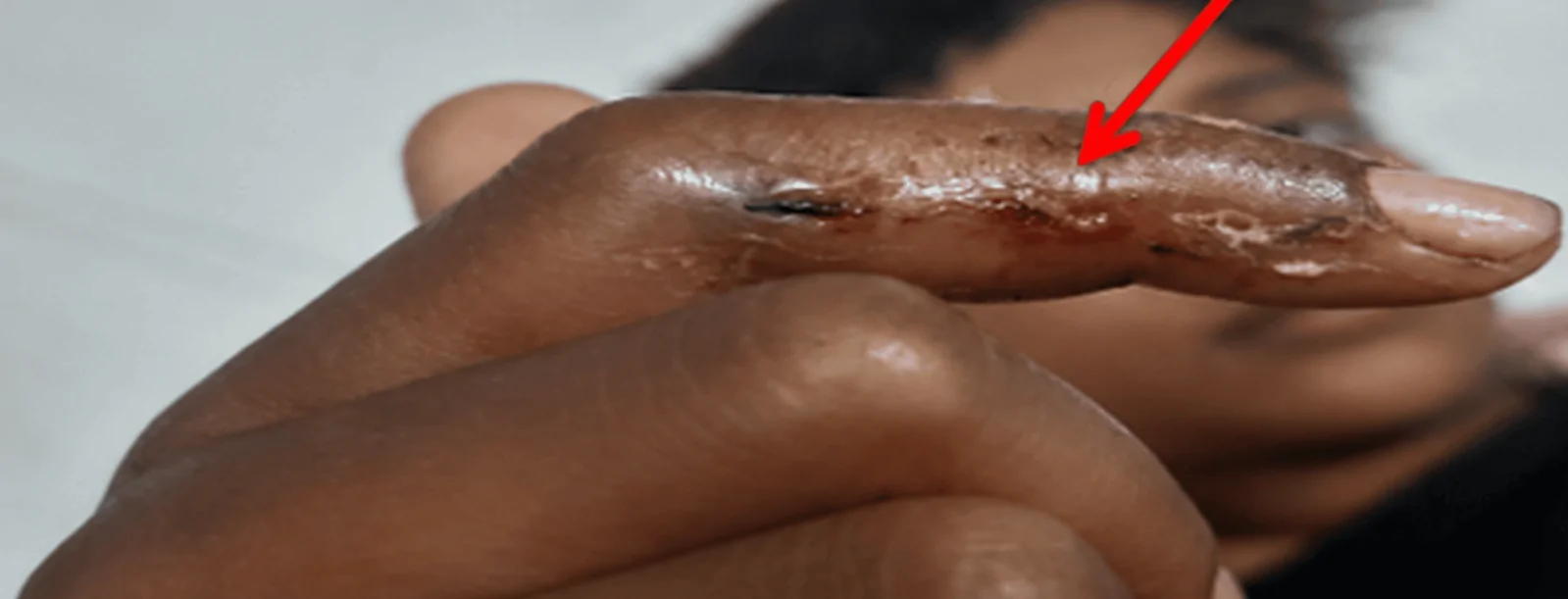
Fang mark over the finger with significant local edema.

Time to presentation and outcomes

Patients were distributed across time groups as follows: Group I (<6 hours): 46.7% (n = 14); Group II (six to 12 hours): 33.3% (n = 10); Group III (12-18 hours): 6.7% (n = 2); Group IV (18-24 hours): 13.3% (n = 4). Overall outcomes were as follows: complete recovery = 73.3% (n = 22), death = 13.3% (n = 4), and LAMA = 13.3% (n = 4).

Mortality was strongly associated with delayed presentation. Out of all four deaths, one death occurred in a patient presenting within six hours. Group IV (18-24 hours) had the highest mortality at 75.0% (3/4), while Group I (<6 hours) had 7.1% (1/14). No deaths occurred in Group II (0/10) or Group III (0/2). Recovery rates were highest in Group I (71.4%, 10/14) and Group II (90.0%, 9/10). The association between delayed presentation and mortality was significant. The association between presentation-time group and mortality (three approaches to LAMA cases) is shown in Table 4.

**Table 4:** Association between presentation-time group and mortality (three approaches to LAMA cases) LAMA: leaving against medical advice

LAMA handling	N	Deaths	Overall exact p†	>12-h vs. ≤12-h mortality	OR (95% CI)‡	p§	Group IV vs. Group I mortality	OR (95% CI)‡	p§
Excluded	26	4	0.014	3/6 (50.0%) vs. 1/20 (5.0%)	13.0 (1.40–120.4)	0.028	3/4 (75.0%) vs. 1/11 (9.1%)	16.3 (1.25–212.6)	0.033
Counted as non-death	30	4	0.009	3/6 (50.0%) vs. 1/24 (4.2%)	15.7 (1.70–144.0)	0.018	3/4 (75.0%) vs. 1/14 (7.1%)	21.0 (1.64–269.0)	0.019
Counted as death	30	8	0.101	3/6 (50.0%) vs. 5/24 (20.8%)	3.55 (0.61–20.6)	0.300	3/4 (75.0%) vs. 4/14 (28.6%)	5.44 (0.60–49.6)	0.245

## Discussion

This study of 30 snakebite patients at AIIMS Raebareli demonstrates a clear and graded relationship between delayed hospital presentation and adverse outcomes. The finding that out of four deaths that occurred in patients, one took place in patient presenting within six hours but initially went to a faith healer, which delayed the treatment, and that three of the four patients in the 18-24-hour group died, which strongly supports the "golden hour" principle in snakebite management.

Our demographic profile aligns with existing Indian literature. The mean age of 28.0 years and male predominance (53.3%) are consistent with reports from tertiary centers across India, where young adults engaged in outdoor labor bear the highest risk of exposure [9-11]. The concentration of cases in the 18-30-year age group (50%) underscores the socioeconomic impact of snakebite, which disproportionately affects the economically productive population, resulting in the loss of livelihood and family distress. The relatively high proportion of female patients (46.7%) may reflect the active involvement of women in agricultural and domestic outdoor activities in rural Uttar Pradesh.

This distribution is similar to the lines of established distribution for bites from lower limbs (53.3%) and nocturnal timing (56.7%). The high prevalence of neurotoxic symptoms (ptosis, dysphagia, and respiratory distress) and 40% upper limb bite rate in our study indicates high exposure to kraits, notably upper limbs at night, which is common in victims sleeping on floors [12].

The socioeconomic gradient is half as 53.3% were from low socioeconomic status and 73.3% had to travel from 15 to 20 km to reach a hospital. This is in keeping with Harrison's definition of snakebite as a disease of poverty [13], where geographical isolation, inadequate transport, and financial limitations come together to delay definitive care. The 13.3% of patients who received harmful first aid (faith healers or incision) further illustrates how traditional practices and inadequate community awareness perpetuate poor outcomes.

The clinical presentation was dominated by neurotoxic features. Universal dyspnoea and drowsiness (100% each), followed by ptosis and slurred speech (90% each), indicate widespread neuromuscular involvement. This pattern is consistent with krait and cobra envenomation, where presynaptic and postsynaptic neurotoxins block acetylcholine release and receptor binding, causing progressive bulbar and respiratory paralysis [14]. The absence of ophthalmoplegia (0%) and low necrosis rate (3.3%) further support a predominantly neurotoxic rather than cytotoxic envenomation pattern in our cohort.

The central finding, i.e., delayed presentation predicts mortality, is biologically plausible and clinically critical. Before ASV administration, circulating venom continues to bind irreversibly to target tissues. Neurotoxic venom blocks neuromuscular transmission; haemotoxic venom triggers consumptive coagulopathy and renal injury [15]. Once bound, ASV cannot neutralize tissue-fixed venom, it only clears circulating unbound venom. Therefore, every hour of delay allows more venom to reach target receptors, progressively reducing the therapeutic window. found a 75% mortality in patients that arrived 18-24 hours after the bite.

Our mortality rate of 13.3% (4/30) is higher than the 2.5-5% reported from centres with better access [16], but comparable to studies from similarly resource-limited settings where delayed presentation is common [17]. The LAMA rate of 13.3% (4/30) is concerning, patients or attendants may lose confidence in treatment when presentation is delayed and disease is advanced, or they may face insurmountable financial barriers despite free hospital care (transport, food, and lost wages). 

Several factors perpetuate delay in our catchment area: poor road connectivity, absence of emergency transport, reliance on traditional healers, low community awareness of envenomation urgency, and referral delays from peripheral centres lacking ASV. Public health interventions must address all these levels simultaneously: community education on immediate hospital transport, ASV stocking at primary health centres, training of medical officers in envenomation recognition, and improved emergency referral networks.

Limitations

This study has several limitations. The sample was small (N = 30) and from a single tertiary centre, limiting statistical power and generalisability, with probable referral bias toward more severe cases. Deaths were few (n = 4) and some groups had only two to four patients, yielding wide confidence intervals and precluding adjustment for confounders; the mortality association should therefore be considered exploratory. Four patients left against medical advice with unknown outcomes, and the significance of the association depends partly on how these cases are handled. The cross-sectional observational design cannot establish causation, and unmeasured factors snake species, envenomation severity, ASV dose and timing, and pre-hospital care may have influenced outcomes. Species was identified in only 70% of cases, and bite and presentation times relied on recall and records subject to imprecision. Larger prospective multicentre studies are needed to confirm these findings.

## Conclusions

Snakebite envenomation remains an important public health concern in rural India. In this hospital-based observational study, delayed presentation was associated with poorer clinical outcomes, with the highest observed mortality among patients presenting 18-24 hours after the bite. However, the small sample size, limited number of deaths, and uncertainty surrounding patients who left against medical advice warrant cautious interpretation of these findings. The results highlight the clinical importance of timely hospital presentation, early administration of ASV when indicated, and appropriate supportive care. Strengthening community awareness, discouraging harmful traditional practices, improving referral pathways, and ensuring timely access to definitive treatment at peripheral healthcare facilities may help reduce preventable morbidity and mortality. Larger prospective studies are needed to determine the independent contribution of presentation delay to outcomes following snakebite envenomation.
